# Zinc Oxide Nanoparticles (ZnO-NPs) Induce Cytotoxicity in the Zebrafish Olfactory Organs via Activating Oxidative Stress and Apoptosis at the Ultrastructure and Genetic Levels

**DOI:** 10.3390/ani13182867

**Published:** 2023-09-09

**Authors:** Sheren A. Al-Zahaby, Mayada R. Farag, Mahmoud Alagawany, Heba S. A. Taha, Maria Vittoria Varoni, Giuseppe Crescenzo, Suzan Attia Mawed

**Affiliations:** 1 Zoology Department, Faculty of Science, Zagazig University, Zagazig 44519, Egypt; sherin@zu.edu.eg (S.A.A.-Z.); samoawad@zu.edu.eg (S.A.M.); 2 Forensic Medicine and Toxicology Department, Faculty of Veterinary Medicine, Zagazig University, Zagazig 44519, Egypt; dr.mayadarf@gmail.com; 3 Poultry Department, Faculty of Agriculture, Zagazig University, Zagazig 44519, Egypt; 4 Genetics Department, Faculty of Agriculture, Zagazig University, Zagazig 44519, Egypt; hebasayedtaha@gmail.com; 5 Department of Veterinary Medicine, University of Sassari, 07100 Sassari, Italy; 6 Department of Veterinary Medicine, University of Bari, 70010 Valenzano, Italy; giuseppe.crescenzo@uniba.it

**Keywords:** zebrafish, zinc oxide nanoparticles (ZnO-NPs), olfactory epithelium, oxidative stress, apoptosis

## Abstract

**Simple Summary:**

Zinc oxide nanoparticles (ZnO-NPs) can exert toxic effects on living organisms. The fish olfactory epithelium is considered the first organ affected by ZnO-NPs, and we demonstrated that a 60-day exposure to ZnO-NPs induced significant malformations of the olfactory rosettes at histological, ultrastructural, and genetic levels in zebrafish, affecting the cellular repairing mechanisms. The present study shows that ZnO-NPs can mediate the cellular oxidative stress and arrest cell growth that induces apoptosis without the ability of cellular regeneration, damaging the olfactory epithelium and affecting fish smell and appetite.

**Abstract:**

Nanotechnology has gained tremendous attention because of its crucial characteristics and wide biomedical applications. Although zinc oxide nanoparticles (ZnO-NPs) are involved in many industrial applications, researchers pay more attention to their toxic effects on living organisms. Since the olfactory epithelium is exposed to the external environment, it is considered the first organ affected by ZnO-NPs. Herein, we demonstrated the cytotoxic effect of ZnO-NPs on the olfactory organ of adult zebrafish after 60 days post-treatment. We opted for this period when fishes stop eating their diet from the aquarium, appear feeble, and cannot swim freely. Our study demonstrated that ZnO-NPs induced significant malformations of the olfactory rosettes at histological, ultrastructural, and genetic levels. At the ultrastructure level, the olfactory lamellae appeared collapsed, malformed, and twisted with signs of degeneration and loss of intercellular connections. In addition, ZnO-NPs harmed sensory receptor and ciliated cells, microvilli, rodlet, crypt, and Kappe cells, with hyper-activity of mucous secretion from goblet cells. At the genetic level, ZnO-NPs could activate the reactive oxygen species (ROS) synthesis expected by the down-regulation of mRNA expression for the antioxidant-related genes and up-regulation of DNA damage, cell growth arrest, and apoptosis. Interestingly, ZnO-NPs affected the odor sensation at 60 days post-treatment (60-dpt) more than at 30-dpt, severely damaging the olfactory epithelium and irreparably affecting the cellular repairing mechanisms. This induced a dramatically adverse effect on the cellular endoplasmic reticulum (ER), revealed by higher CHOP protein expression, that suppresses the antioxidant effect of Nrf2 and is followed by the induction of apoptosis via the up-regulation of Bax expression and down-regulation of Bcl-2 protein.

## 1. Introduction

The future insight into nanomaterials utilization has grown considerably. Along with silicon dioxide nanoparticles, zinc oxide nanoparticles (ZnO-NPs) are considered one of the most applied nanomaterials in biomedical industries [1,2,3,4,5,6]. The bio-safety of ZnO-NPs is still a controversial issue since increasing studies assumed that they are biocompatible and could be applied to biomedical materials [7,8,9,10]; at the same time, others indicated the toxicological influences of ZnO-NPs on living species from bacteria to humans [11,12,13,14,15,16,17,18,19,20].

Zebrafish have gained great importance in developmental genetics, aquatic toxicology, and neuroscience research [21,22] due to their unique physiological aspects, such as external fertilization, rapid development, and rapid recovery rate from damage [23]. Although extensive research has been conducted on the zebrafish at the embryonic and larval stages [24,25], studies of ZnO-NPs’ influence on the adult stage are still limited.

For most living creatures, olfaction is one of the most important ways of interacting with the environment. It is one of the oldest senses, permitting organisms with receptors for the odorant to identify the surrounding environment and territory [26,27], contributing to food finding, predator escaping, and mating in wildlife [27]. The olfactory system is known for its role in associative behaviors mediated by odorants sensed in the olfactory mucosa and olfactory receptors in the olfactory epithelium of the nasal cavity, so it plays a fundamental role in the daily life of all animal species [26].

Olfactory dysfunction has received increased attention recently because it is a common age-related disease associated with some pathologies [1,2,28]. These pathologies include viral infection and the intake of toxic substances related to the progress of olfactory dysfunction [29]. Other studies have also demonstrated that olfactory dysfunction is an independent risk factor, even in mortality [30,31]. This is because the peripheral olfactory organs are open to the environment; therefore, they are always at risk of being injured by extrinsic pathogens and chemicals, unlike the peripheral structures of most other sensory systems [27].

Indeed, few studies referred to the toxic effects of nanoparticles on the olfactory bulb and sensory epithelium, especially in teleost. Exposure to copper nanoparticles in rainbow trout led to detrimental effects, including oxidative stress and immune suppression [32], silver nanoparticles induced a toxic effect on the olfaction of Crucian carp [33], and ZnO-NPs exhibited a toxic effect on the rat olfactory system [34].

Therefore, the main goal of the present work was to study the hazardous effect of ZnO-NPs on the olfactory sensory cells in the zebrafish model via investigating the histological and ultrastructural alterations but also the changes in the mRNA expressions of antioxidant-, stress-, and apoptotic-related genes to clarify the mechanisms of ZnO-NPs toxicity.

## 2. Materials and Methods

### 2.1. Zebrafish Husbandry and ZnO-NPs Exposure

Adult zebrafish (*Danio rerio*) were bought from a local fish supplier (Cairo, Egypt). The fish supplier reared only wild-type zebrafish in large glass tanks (1270 mm × 610 mm × 457 mm) with known reared dates. Adult males (n = 90; average weight 0.62 ± 0.21 g, 6 months old) were acclimatized for two weeks before the experiment. During the acclimatization and throughout the investigation, fishes were kept in aerated de-chlorinated tap water at 27.5 ± 1 °C in glass aquaria (80 × 40 × 30 cm, water capacity 60 L) 14 h light:10 h dark, pH 6.7 ± 0.2 and dissolved oxygen 6.3 ± 0.5 mg/L. The fish were fed twice daily on *Artemia nauplii* (hatched shrimp eggs) according to well-established protocols [35]. Acclimatized fishes were randomly divided into two experimental groups (n = 45/group), each in triplicate (15 fish in each replication in separate tanks). The first group served received no treatment (control group), while the second group was exposed to 1/5th of the estimated LC_50_ of ZnO-NPs (0.69 mg/L) in the water daily for 60 days (treated). All the animal procedures used in this study were approved by the Institutional Animal Care and Use Committee of the Zagazig University, Zagazig, Egypt (Approval number: ZU-IACUC/1/F/213/2023).

The synthesis and characterization of ZnO-NPs were previously described in Mawed et al. (2022), where the UV-VIS spectroscopy results of the characterization of ZnO-NPs show the maximum peak at 340 nm [36]. TEM analysis reveals a spherical shape with an average size of 108 nm. The net surface charge is −33 mV based on the data from the zeta potential analysis. The hydrodynamic size is 89 nm based on the DLS analysis. The LC_50_ of ZnO-NPs was estimated in our laboratory and found to be 3.48 mg/L.

### 2.2. Scanning Electron Microscopy (SEM) Preparations

The sacrificed adult zebrafish heads were dissected under a stereoscopic binocular microscope (ZEISS LuMAR.V12, Oberkochen, Germany) to expose olfactory rosettes in the bottom of the olfactory chamber; then, they were quickly fixed in vivo by perfusion with a mixture solution (2% formaldehyde, 1.25% glutaraldehyde in 0.1 M sodium cacodylate buffer, pH 7.2) for about 24 h at 4 °C. After fixation, the rosettes, which were lifted in their natural position, were rinsed in the same buffer for about 10 min and subjected to post-fixation in 1% osmium tetroxide in 0.1 M cacodylate buffer (pH 7.2) for 2 h. The setting rosettes were washed with the same buffer and dehydrated through a graded acetone series, which was followed by amyl acetate. The dehydrated rosettes were then dried using a Critical Point Dryer (Hitachi Ltd., Tokyo, Japan) [37]. The dried specimens were then fixed to stubs with the colloidal carbon and coated by gold–palladium in a sputtering device. Then, they were examined and photographed by a JEOL SEM device (Faculty of Science, Alexandria, Egypt) at an accelerating voltage of 15 kV, a working distance of 5.6–6.6 mm, and a standard acquisition resolution of 1536 × 1024.

### 2.3. Transmission Electron Microscopy (TEM) and Semi-Thin Sections Preparations

The fixed olfactory rosettes were cut into small pieces and then post-fixed for 2 h at room temperature in the same glutaraldehyde solution and 1% osmium tetroxide. Rosette pieces were then dehydrated in a graded ethanol series and embedded in an Epon–Araldite mixture. Then, using a Reichert ultra-microtome, ultrathin sections of 1.5 mm were cut and stained with toluidine blue contrasted in a 50% alcohol–uranyl acetate solution and lead citrate with a transmission Philips EM 400 electron microscope (Philips, INCAEDX, Oxford, UK) [30]. The semi-thin sections after toluidine staining were examined and photographed (Faculty of Science, Alexandria, Egypt).

### 2.4. Histological Assessments by Light Microscope

The newly separated olfactory rosettes were fixed in 10% formalin. After 24 h, rosettes were appropriately dehydrated through an ascending series of ethyl alcohols, cleared with xylene, and embedded in paraffin wax. Tissue sections at 5 μm were prepared using a microtome (Leica Model, RM2125 Biosystems, Deer Park, NY, USA) and stained with hematoxylin and eosin (H&E) to be examined and photographed by light with a PANNORAMIC MIDI I (Digital Slide Scanners MIDI, 3D HISTECH Company, Budapest, Hungary) [38].

### 2.5. Total RNA Extraction and Quantitative Real-Time PCR (qRT-PCR)

Total RNA was isolated from the control and treated olfactory rosettes (n = 10/group) using TRIzol reagent according to the manufacturer’s instructions (Life Technologies, Carlsbad, CA, USA). RNA purity and concentration were evaluated using gel electrophoresis and the spectrophotometer (METTLER, TOLEDO, Mississauga, ON, Canada). First, 1 μg from the isolated RNA was reverse transcribed into cDNAs by PrimeScript TM RT reagent Kit with gDNA Eraser (Stratagene, Takara, Shiga, Japan). Quantitative real-time PCR was performed on the MSLPCR30 Thermal Cycler system (Biobase Biozone Co., Ltd., Shaanxi, Guangdong, China) with thermal cycling conditions comprised denaturation at 95 °C for 1 min followed by 40 cycles of 95 °C for 10 s and then 60 °C for 20 s and 72 °C for 30 s. The mRNA expressions of the studied genes were normalized to β-actin, and the primers used in the study are listed in Table 1.

### 2.6. Protein Extraction and Western Blot Analysis

Total protein was extracted from homogenates of 8 frozen olfactory organs (stored at −80 °C) for each experimental group, including control and ZnO-NPs treated for 30 and 60 days. Western blot was performed according to a previous report [36]. After the processes of electrophoresis and electro-transferase, Nylon Fluoride membranes (Millipore, Burlington, MA, USA) were cut at the desired band of protein marker and then washed by Tris-Buffered Saline Tween (TBST) for 15 min; then, they were blocked against 5% BSA (Bovine Serum Albumin, AUG pharma, Giza, Egypt) for 1 h in room temperature. Nylon membranes were blotted with primary antibodies overnight at 4 °C. Primary antibodies were kindly provided from the National Research Central (El-Dokii, Giza, Egypt), and procedures were performed according to the manufacturer’s instructions: Nrf2 antibody (N2C2) (1:500 dilution, Internal Cat. No. GTX103322, Gene Tex, Alton, IL, USA), rabbit anti-Chop protein (GADD153) (1:400, G6916, Sigma Aldrich, Taufkirchen, Germany), Bax polyclonal antibody (1:5000 dilution, 50599-2-lg, proteintech, Manchester, UK), Becl-2 antibody N1N2 (1:1000, GTx100064, GeneTex, Alton, IL, USA) and rabbit anti-β-actin was used for normalization (1:1000 dilution, Cell signaling, 4967S, Inc., Global Headquarters, USA), respectively. The next day, blots were probed with a secondary antibody (HRP-conjugated, Abcam, Cambridge, UK) and then visualized by ECL Western blot detection reagents. Band scanning and protein area quantifications were detected by NIH software Image J. 1.51 k; Java 1.6.0_24.

### 2.7. Statistical Analysis and Graphs Preparation

Statistical analysis was performed using SPSS. Data were analyzed by Student’s *t*-test when comparing two groups and one-way ANOVA when comparing more than two groups. All data are presented as the means ± standard error of the mean and were first checked for normality using the D’Agostino–Pearson normality test. Histological image quantification and analysis were calculated by the 3D Histech Quant Center (3DHISTECH Budapest, Hungary). For protein band quantification, images were analyzed using Image J software (1.51k, Java1.6.0., National Institutes of Health, Bethesda, Maryland, USA). All plots and graphs were designed using Graph Pad Prism 8 package (GraphPad Inc., La Jolla, CA, USA). The significance was given as (* *p* < 0.05; ** *p* < 0.01; *** *p* < 0.001) [39,40,41,42,43,44,45].

## 3. Results

### 3.1. Macrostructure of Zebrafish Olfactory Organ

**Figure 1:**
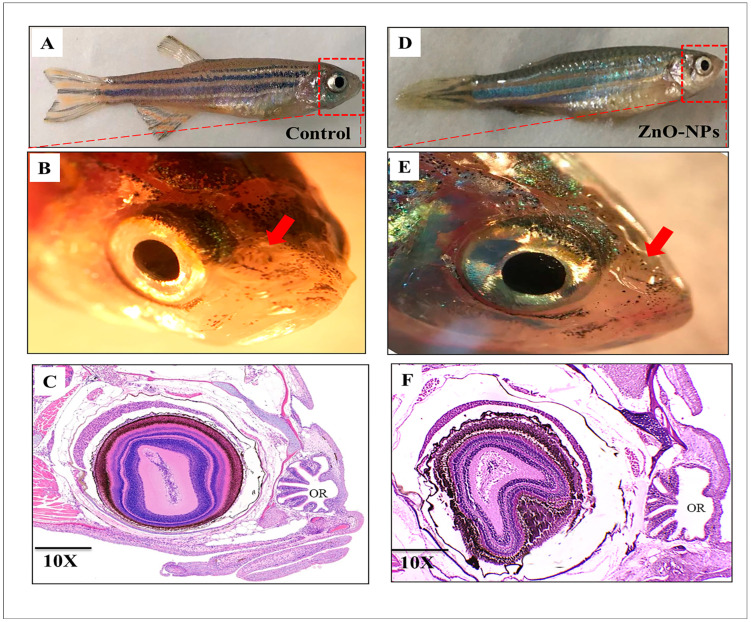
Gross morphology of the adult zebrafish (*Danio rerio*) olfactory rosette and main olfactory system structures: (**A**) Control zebrafish, (**B**) Lateral view of a dissected olfactory rosette (OR) of control fish (red arrow), (**C**) H&E histological assay of a longitudinal section of the whole control OR showing some lamellae; (**D**) ZnO-NPs treated zebrafish, (**E**) Lateral view of a dissected OR of treated fish (red arrow), and (**F**) H&E histological assay of a longitudinal section of the whole treated OR showing some lamellae.

Anatomically, zebrafish have two nasal cavities (olfactory chamber) anterodorsally positioned and slightly close to the orbits and mouth; in a parasagittal section at the orbital level, each nasal cavity is covered by a boat sail-shaped skin flap that outlines the funnel-shaped inlet and outlet. Through these openings, water conveying odorants circulates via the olfactory chamber to submerge the sensory structure (olfactory rosette) sitting on the bottom. Longitudinal sections show normal olfactory rosette organization in the control fish (Figure 1A–C). On the other hand, the ZnO-NPs treated group showed a malformed olfactory rosette with stacked lamellae (Figure 1D–F).

### 3.2. ZnO-NPs Alter the Histological Architecture of the Olfactory Rosette

**Figure 2:**
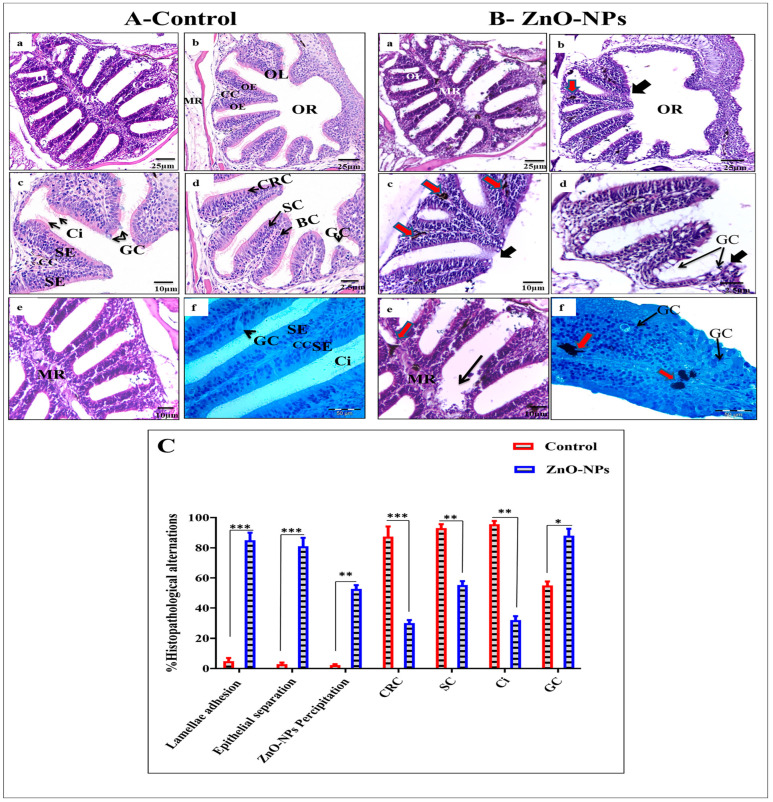
H&E histological assay of (**A**) the control and (**B**) ZnO-NPs-treated zebrafish olfactory rosettes: (**A**(**a**)) horizontal section of the whole control olfactory rosette (OR), olfactory lamellae (OL), median raphe (MR); (**A**(**b**)) transverse section of the whole control OR, OL, MR, and central core (CC); (**A**(**c**)) magnified part of the OR showing CC, sensory epithelium (SE), ciliated receptor cells have darken spindle shape (CRC), cilia of the ciliated non-sensory cell (Ci), and goblet cell (GC); (**A**(**d**)) magnified part of the olfactory rosette showing supporting cell (SC) and basal cell (BC); (**A**(**e**)) magnified portion of the olfactory rosette showing CC; (**A**(**f**)) photomicrographs of the semi-thin section stained with toluidine blue of the olfactory lamella showing CC, SE, Ci, and GC (black arrow); (**B**(**a**)) horizontal section of the whole treated OR, collapsed OL, necrosis in MR; (**B**(**b**,**c**)) transverse section of the whole treated olfactory rosette showing swollen and detached olfactory epithelium (black arrow) and precipitation of ZnO-NPs in the olfactory epithelium and the central core (red arrow); (**B**(**d**)) magnified part of the olfactory rosette showing a large number of GC (black arrow); (**B**(**e**)) magnified portion of the olfactory rosette showing lamellar destruction (black arrow) and precipitation of ZnO-NPs (red arrow); (**B**(**f**)) photomicrographs of the semi-thin section of the olfactory lamella showing a large number of GC (black arrow) and precipitation of ZnO-NPs (red arrow). (**C**) Statistical analysis for three different sections from the OR of the control and treated group showing histopathological alterations after ZnO-NPs treatment. The results are the mean ± SEM.* *p* < 0.05, ** *p* < 0.01, *** *p* < 0.001.

Light microscope observations for the control group revealed the normal histological architecture of the olfactory rosette. In Figure 2A, the arranged olfactory lamellae radiating from the midline raphe are represented. Each olfactory lamella appears to be covered on both sides by a pseudostratified columnar epithelium that encloses a central lamellar stromal sheet, the central core. This central core comprises loose connective tissues of collagenous fibers comprising a network of blood capillaries and nerve fibers which run from the olfactory epithelium to the olfactory bulb. The olfactory epithelia on both sides of the central core are of unequal thickness (Figure 2A(b–d)). The sensory epithelium comprises spindle-shaped (bipolar neuron) ciliated receptor cells with oval nuclei and cylindrical non-sensory supporting cells arranged in alternate rows besides rounded basal cells and goblet mucous cells. However, besides the last two types of cells, basal and mucous goblet cells, the non-sensory epithelium embraces round to cubical indifferent epidermal cells and some cylindrical supporting cells, as shown in Figure 2A(c,d). The basal cells are relatively small and spherical, with rounded nuclei scattered in the whole epithelium’s deeper part just above the basal lamina. They are a reservoir for forming receptors and supporting cells as they migrate toward the upper part of the olfactory epithelium. The goblet mucous cells are scattered all over the superficial layer of the olfactory epithelium, particularly in the non-sensory area (Figure 2A(c,d,f)). They vary in size and shape according to the activity; most are oval with basal nuclei.

On the other hand, the ZnO-NPs treated group showed an obvious disorganization of the lamellar arrangement of the treated fish. Olfactory lamellae appeared collapsed, malformed, and twisted with signs of degeneration and loss of intercellular connections (Figure 2B(a,b)). The olfactory epithelium appeared swollen with many holes between the central core and detached from the basal lamina (Figure 2B(c,d)). It was also evident that the cilia of different olfactory sensory and non-sensory were decreased in number, adherent, and stuck together. Also, goblet mucous cells were hyperactivated (Figure 2B(d,f)) with a higher precipitation of ZnO-NPs in the central core of the olfactory lamellae and appeared as large black accumulations (Figure 2B(c,e,f)). Statistical analysis of histopathological alterations in the olfactory rosette for both control and ZnO-NPs treated zebrafish indicated high ZnO-NPs precipitation in the treated fishes, lamellar adhesion, epithelial separation, and goblet cells were also well distributed. At the same time, the percentages of ciliated receptor cells, supporting cells, and distributed cilia were scarcely observed (Figure 2C).

### 3.3. ZnO-NPs-Treated Rosette Exhibited Lamellar Sensory Area Destruction at the Scanning Electron Microscope (SEM) Level

**Figure 3:**
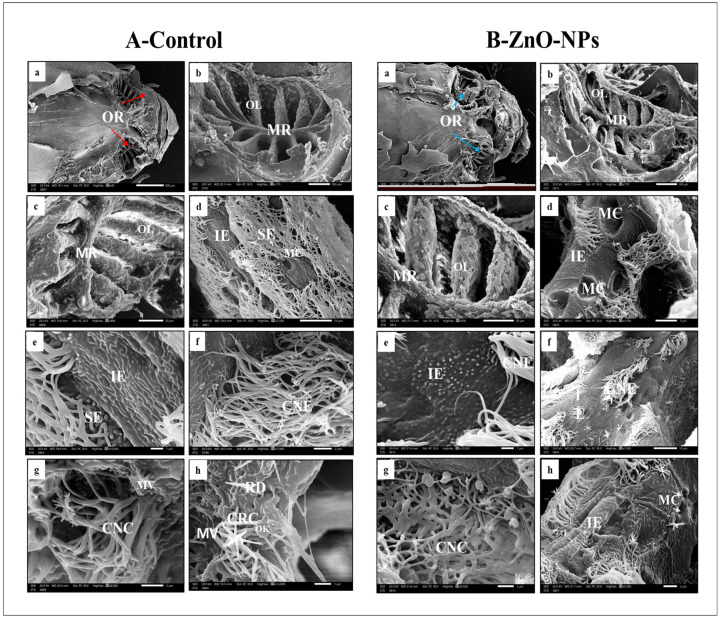
Scanning electron microscopy observations of the epithelial surface of (**A**) the control and (**B**) ZnO-NPs zebrafish olfactory rosette: (**A**(**a**)) scanning electron micrograph of the head of an adult zebrafish with two olfactory rosettes (ORs) (red arrows); (**A**(**b**)) olfactory rosette located in the nasal cavity, with olfactory lamellae (OL) arranged on both sides of the midline raphe (MR); (**A**(**c**)) higher magnification of the OL and the MR; (**A**(**d**,**e**)) sensory epithelium (SE) and indifferent epithelium (IE) are strictly separated; (**A**(**f**)) lamella is covered with ciliated non-sensory epithelium (CNE); (**A**(**g**)) cilia of the ciliated non-sensory cell (CNC); (**A**(**h**)) the surface of the sensory epithelium shows ciliated receptor cells (CRC) with olfactory knobs (OK), microvillous receptor cells (MV), and rod cells (RD); (**B**(**a**)) scanning electron micrograph of the head of an adult zebrafish with two treated OR (blue arrows); (**B**(**b**,**c**)) the olfactory rosette stunted appearance with preserved OL and the MR; (**B**(**d**)) numerous pores of mucous cells (MC), the IE without micro ridges; (**B**(**e**)) IE without micro-ridges; (**B**(**f**–**h**)) cilia of CNC fused with a decreased number.

On the bottom of each nasal cavity of the control group, the olfactory rosette appears as an oval-shaped multi-lamellar sensory structure. Its olfactory lamellae are lined up on a wide midline raphe to support the lamellae, which radiate outward and extend toward the periphery of the nasal cavity, increasing in size as it goes from the rostral to caudal direction. The young lamellae are rostral built at both sides of the midline raphe; these olfactory lamellae range from 12 up to 15 (Figure 3A(a–c)). On higher magnification, the olfactory epithelia covering each olfactory lamella are separated into non-sensory and sensory epithelia (Figure 3A(d,e)). The edge of each lamella is covered with the ciliated non-sensory epithelium of ciliated non-sensory cells organized in a valleys-like system that extends to the upper edges of the lamellae as well as indifferent epithelium with apparent micro-ridges and goblet mucous cells observed in between (Figure 3A(f)). However, the medial part of each side of the lamellae encloses the olfactory sensory epithelium continuously planned with its receptor cells. It also covers the intervening areas between every two successive lamellae at the midline raphe.

On SEM, we could detect three receptor cell types based on the structure and shape of their apical dendrites, whether ciliated, microvillar, or rod-shaped (Figure 3A(g,h)). The sensory cells can be organized by scanning microscopy into:

i. Ciliated receptor cells, their dendritic part protrudes slightly beyond the supporting cells’ boundary, forming a small terminal swelling hillock-like apex, the olfactory knob. From this knob, six to eight long cilia or flagella arise radially projecting into the lumen of the olfactory cavity (Figure 3A(h)).
ii. Microvillous receptor cells with apical surfaces full of numerous shorter microvilli protruding into the olfactory space (Figure 3A (g,h)).
iii. Rod cell shows rod-like projection and protrudes from its free surface by an olfactory knob. Rod neurons can be observed randomly between the ciliated sensory cells (Figure 3A(h)).

Contrarily, the olfactory rosette of the zebrafish treated with ZnO-NPs showed a stunted appearance with preserved olfactory lamellae and degenerated olfactory rosette (Figure 3B(a–c)). The long cilia of ciliated non-sensory cells were decreased in number, thinned out, curled, and fused. This was observed mainly in the outer distal region of the lamella with massive mucus secretion given by the activated mucous cells with wider pores compared to the normal non-treated fish (Figure 3B(d)). The micro-ridges of the indifferent epithelium lost their linear appearance and sometimes disappeared entirely in some areas (Figure 3B (e,f)).

Concerning the sensory epithelia, they were extensively damaged, with almost complete loss of cilia and microvilli of all receptor cells to the extent that some of their knobs appeared utterly naked, and some with a rod-like extension, the ciliated and microvillous neurons had been hardly detected (Figure 3B(f–h)).

### 3.4. ZnO-NPs Induce Sensory Cell Apoptosis and Cytoplasmic Organelles Destruction at the Transmission Electron Microscope (TEM) Level

**Figure 4:**
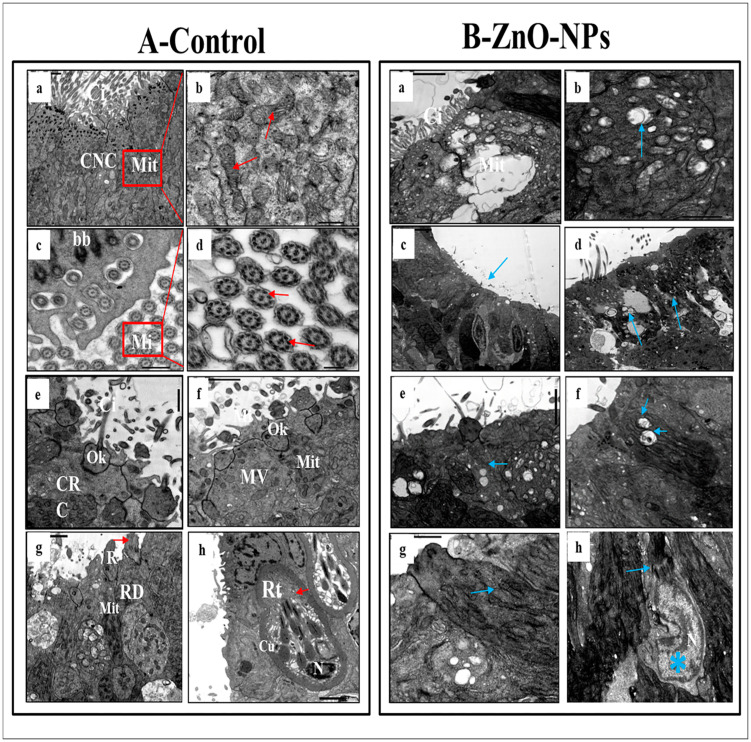
Transmission electron micrographs of (**A**) the control and (**B**) ZnO-NPs-treated zebrafish olfactory epithelium: (**A**(**a**)) olfactory epithelium consisting of ciliated non-sensory cells (CNC) with cilia (Ci) and mitochondria (Mit); (**A**(**b**)) magnified part of ciliated non-sensory cells showing Mit; (**A**(**c**,**d**)) cross-section of cilia exhibits nine pairs of outer microtubules (Mi) and two central ones (red arrow), basal body (bb); (**A**(**e**)) magnified view of ciliated receptor cells (CRC) showed an olfactory knob (Ok) bearing cilia (Ci). (**A**(**f**)) magnified view of microvillous receptor cell (MV), which showed an Ok bearing short microvillar processes (Mi) on its apical surface and Mit; (**A**(**g**)) magnified view of rod cell (RD) shows parallel oriented microtubules (red arrows) in rod-like cilia (R) and Mit. (**A**(**h**)) the rodlet cell (Rt) is identified by the thick cuticle (cu) and its typical rodlets (red arrows) and nucleus (N); (**B**(**a**–**d**)) olfactory epithelium consisting of ciliated non-sensory cells with decreased number of Ci and Mit swelling and vacuolation (blue arrow); (**B**(**e**–**g**)) the cilia, microvilli, and rods of all olfactory neurons injured mitochondria swollen and vacuolated (blue arrow); (**B**(**h**)) rodlet cell with hypertrophy nucleus (blue star) and detached apical rodlets (blue arrow).

TEM observations highlighted the olfactory epithelium of the control group into ciliated non-sensory cells and seven distinct types of olfactory sensory receptor cells. All these cells are closely crowded, including ciliated receptor cells, microvillous receptor cells, rod cells, rodlet cells, crypt cells, Kappe cells, and pear-shaped cells in addition to the supporting cells, the small rounded basal cells, and the mucous goblet.

i. Ciliated non-sensory cells are club-shaped with a narrow, deep surface and a relatively flat, broad, free surface. They do not have dendrites and axons but have numerous long rootlet cilia arising as a tuft oriented in the same direction from the cell’s surface (Figure 4A(a)). At the most apical cytoplasmic region, plentiful oval or rounded mitochondria are found without preferential orientation (Figure 4A(a,b)). Each cilium’s basal structure comprises a pair of centrioles and one rootlet (Figure 4A(c)). These cilia are kino-cilia, showing a typical axonemal pattern (9 + 2) of microtubules (mi) (Figure 4A(c,d)).
ii. Ciliated receptor cells are long, slender-shaped bipolar sensory cells (neurons). Their most basally located somata are highly granulated cytoplasm packed with elongated or rounded mitochondria. A basal axonal process penetrates the basal lamina, and apical long dendritic processes extend toward the surface of the olfactory epithelium. These processes (cilia) randomly arise from an olfactory knob. Each cilium has a basal body without any rootlets but is associated with centrioles with several neuro-filaments present just beneath the plasma membrane of the olfactory knob (Figure 4A(e)).
iii. Microvillous receptor cells are also elongated bipolar cells (neurons), their bodies featuring short dendrites and visible nuclei located at intermediate depths of the olfactory epithelium. Their apical surface is provided with a tuft of shorter dendrites, microvilli, which project radially outwards in the olfactory lumen from a concave olfactory knob under the surface level of the adjacent supporting cells. Some basal bodies are arranged in two rows at the microvilli’s base. Numerous elongated or rounded mitochondria are scattered in the cell cytoplasm (Figure 4A(f)).
iv. Rod receptor cells are elongated bipolar cells (neurons) but differ from the other receptor cells in having a single thick cilium, rod-like projection, so they are known as rod receptor cells. This rod-like projection extends from a knob-like apex and has a pair of basal bodies at the base of its axonemal microtubules. As in all receptor cells, the cell body of a rod cell is fully packed with mitochondria scattered within its granulated cytoplasm (Figure 4A(g)).
v. Rodlet cells are ovoid-shaped cells enclosed by a distinctive thick cuticula-like wall, found against the inner aspect of the cell plasma membrane, with a narrow apex pore. So, there is not any junction with neighboring cells. These cells have a typical animal cell structure with a basally located nucleus of various forms and shapes. Their cytoplasm is crowded with rounded to elongated vesicular mitochondria as well as few vesicular vacuoles and free ribosomes in addition to clusters of its most striking club or rod-shaped electron-opaque vesicles, rodlets (Figure 4A(h)).

vi. Crypt cells are olfactory sensory neurons (OSNs) with elongated pear-shaped somata found near the apical surface of the sensory epithelium of examined zebrafish.

**Figure 5:**
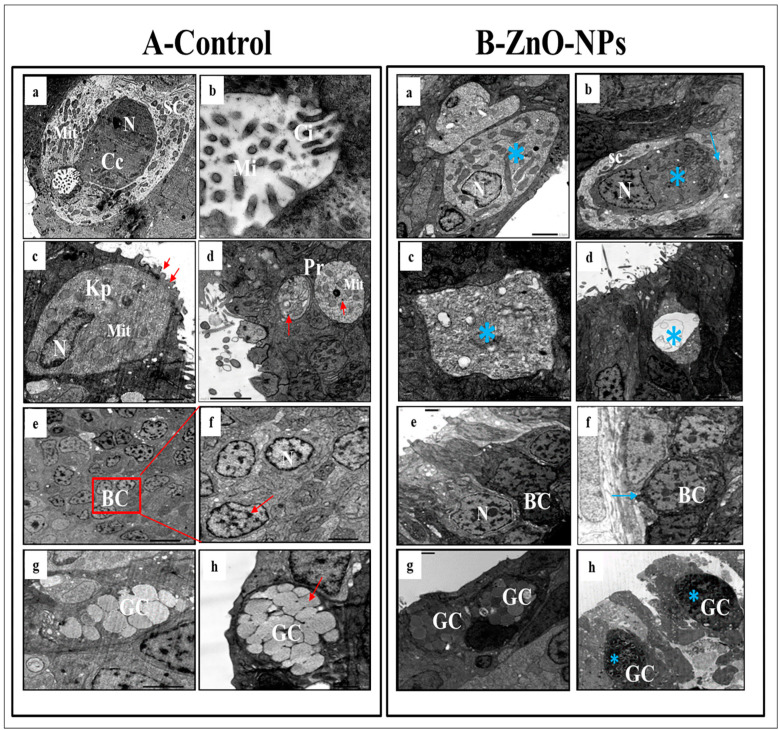
Transmission electron micrographs of the degenerated sensory cells of (**A**) the control and (**B**) ZnO-NPs-treated zebrafish olfactory epithelium: (**A**(**a**)) crypt cell (Cc) with in-sunk cilia, the nucleus (N) in the lower portion of the cell, supporting cell (SC) surrounding the crypt cell and mitochondria (Mit); (**A**(**b**)) higher magnification of the upper portion of a crypt cell, (Cc) showing cilia (Ci) and microtubules (Mi). (**A**(**c**)) the Kappe cells (KP), the nucleus (N) in the basal part of the cell, micro-ridges upper part (red arrows), and mitochondria (Mit); (**A**(**d**)) pear-shaped cell (Pr), the cytoplasm with mitochondria (Mit) and ribosomes (red arrows); (**A**(**e**,**f**)) basal cells (BC, red arrow) with N; (**A**(**g**,**h**)) goblet cells (GC); (**B**(**a**,**b**)) crypt cell (Cc) showing the disappearance of cilia, elongated mitochondria (blue star), and the surrounding supporting cells (SC) thinned out; (**B**(**c**,**d**)) the Kappe and pear-shaped cells that have lost their distinct shape (blue star); (**B**(**e**,**f**)) basal cells (BC) increased in size, and with the nucleus (N) enlarged; (**B**(**g**,**h**)) hyper-activated goblet cells (GC) with retention of a large amount of ZnO-NPs (blue star).

Although crypt cells occur regularly in all lamellae, their absolute number is low and not as high as that of ciliated and microvillous OSNs. They bear cilia and submerged microvilli (Figure 5A(a,b)). These cilia, as those of ciliated OSNs, show the typical (9 + 2) axonemal pattern of microtubules (Figure 5A(b)); the cytoplasm of these crypt cells is electron-dense, packed with elongated or rounded mitochondria and free, abundant ribosomes, but its nucleus fills about one-third of the cell body (Figure 5A(a)). These crypt cells are surrounded by one or two specialized electron-lucent supporting cells bearing micro-ridges but do not have cilia.

vii. Kappe cells are found in the most apical epithelial positions close to the olfactory lumen. Their cell bodies are elongated pear-shaped, similar to crypt cells, but their inward superficial cap apical end is fortified with few cilia and microvilli. Their less flattened nuclei are positioned in the basal part of the cell body and swim in the ground cytoplasm with fully packed rounded mitochondria (Figure 5A(c)).
viii. Morphologically, a pear-shaped cell is similar to Kappe cells but rounded and variable in size. It is located in the apical part of the epithelium, and its cytoplasm has numerous rounded mitochondria-free ribosomes (Figure 5A(d)).
ix. Basal cells are intermingled with the basal portion of the other cell types in the olfactory epithelium. They are small polyhedral cells having a distinct globular shape and darkly stained centrally located nuclei. Basal cells are grouped and interposed by the bases of the sensory and non-sensory supporting cells, forming a discontinuous layer in the deeper part of the epithelium just above the basal lamina and did not reach the free surface of the epithelium (Figure 5A(e,f)).
x. Goblet mucous cells are restricted to the non-sensory epithelium. They are oval-shaped, having basally located nuclei, and about two-thirds of the cell body is filled with large mucous granules. They are surrounded by ciliated non-sensory or epidermal cells bearing micro-ridges. Mature goblet cells secrete their mucous granules into the lumen of the olfactory cavity over the non-sensory epidermal cell surface (Figure 5A(g,h)).

Conversely, in ZnO-NPs-treated fishes, the cilia emitted from the ciliated non-sensory cells appeared shrunken and decreased in number. The mitochondria showed signs of partial or complete degeneration through swelling and vacuolation; tight junctions between different cells were diminished and rarely noticed (Figure 4B(a–d)).

Cilia, microvilli, and rods of all olfactory ciliated microvillus and rod cells (neurons) were significantly injured with large cytoplasmic empty vacuoles (Figure 4B(e–g)), respectively. The mitochondria in all these sensory cells (neurons) were swollen and vacuolated compared with the control siblings. They had a clear matrix and lost their electron-dense deposits with destructive changes in their cristae (Figure 4B(d–f)). Furthermore, the appearance of swollen or filamentous mitochondria in the rod cells was evident (Figure 4B(g)).

Moreover, the rodlet cell lost its thick cuticle-like characterized by degenerated rodlets and hypertrophy nucleus with an irregular crescent shape containing detached chromatin as a sign of oncocytic changes (Figure 4B(h)).

Alongside, the crypt cells lost their distinctive shape. The nucleus appeared irregular and highly condensed; dark chromatin masses were observed but with a continuous nuclear envelope. Their mitochondria were swollen and vacuolated with stacked cilia and microvilli, and the surrounding supporting cells were squeezed and decreased in size (Figure 5B(a,b)).

The Kappe cells and pear-shaped cells appeared empty and lost their distinct shape. The nucleus seemed shrunken with decreased chromatin without a nuclear envelope; the mitochondria were swollen and vacuolated (Figure 5B(c,d)). Basal cells showed a compensatory increase in size; the nucleus was enlarged with condensed chromatin surrounded by a distinct nuclear membrane (Figure 5B(e,f)). The goblet mucous cells+ showed an increased number and became hyper-activated. Their secretions covered the olfactory epithelium’s surface with many ZnO-NPs retaining the black stain on the goblet granules (Figure 5B(g,h)).

### 3.5. ZnO-NPs Induce Apoptosis in the Olfactory Epithelium via Mediating Oxidative Stress and DNA Damage

**Figure 6:**
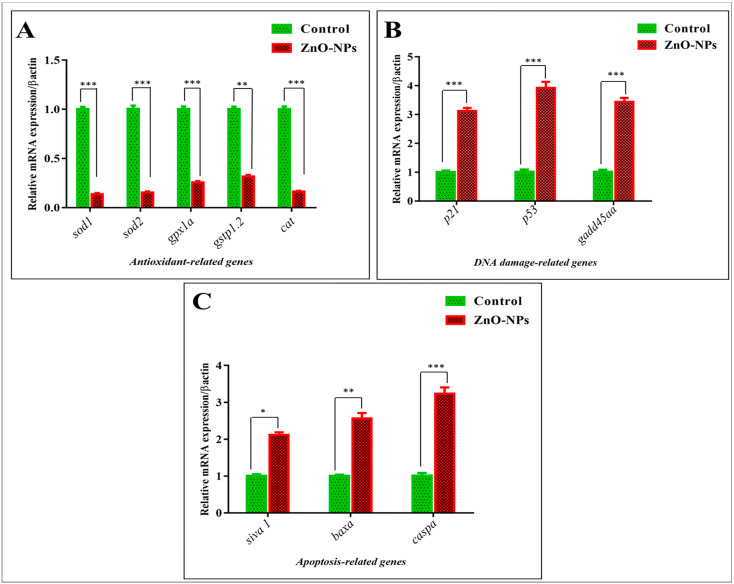
ZnO-NPs-induced cellular apoptosis via oxidative stress and DNA damage at the genetic level: (**A**) mRNA expression evaluated by qRT-PCR for antioxidant-related genes; (**B**) mRNA expression evaluated by qRT-PCR for DNA damage-related genes; (**C**) mRNA expression evaluated by qRT-PCR for apoptosis-related genes. The results are the mean ± SEM.* *p* < 0.05, ** *p* < 0.01, *** *p* < 0.001.

To detect the underlying mechanisms of ZnO-NPs cytotoxicity effect on the zebrafish olfactory organs, the mRNA expression of antioxidant, DNA damage, and apoptosis-related genes was evaluated by qRT-PCR [44] to support our TEM observations between the control and ZnO-NPs treated groups. It has been observed that ZnO-NPs alter mitochondrial structure and function, resulting in the generation of excessive reactive oxygen species (ROS) that weaken the antioxidant enzyme activity, altogether placing the olfactory cells at oxidative stress risk [46,47,48,49]. Accordingly, the mRNA expression of antioxidant-related genes, including *sod1*, *sod2*, *gpx1a*, *gstp1.2*, and *cat*, was down-regulated in the treated olfactory rosette compared with the control one (Figure 6A). In addition, the ROS mediate genotoxicity, which results in chromosomal alteration and DNA damage [50,51], and this is revealed by the up-regulation of cyclin-dependent kinase inhibitor (CDKN1A) or *p21*, tumor suppressor protein *p53*, and growth arrest and DNA damage-inducible alpha a *gadd45aa*, all of which play an essential role in inhibiting DNA replication and subsequently induce cell cycle arrest, apoptosis, and senescence (Figure 6B). In the present study, cellular apoptosis was revealed in the TEM of the treated rosette by destroying the sensory and supporting cells’ cytoplasmic components. The latter observations were confirmed by the mRNA up-regulation of pro-apoptotic related genes, including *siva1*, *baxa*, and *caspa*, in the treated group compared with the control group (Figure 6C).

**Figure 7:**
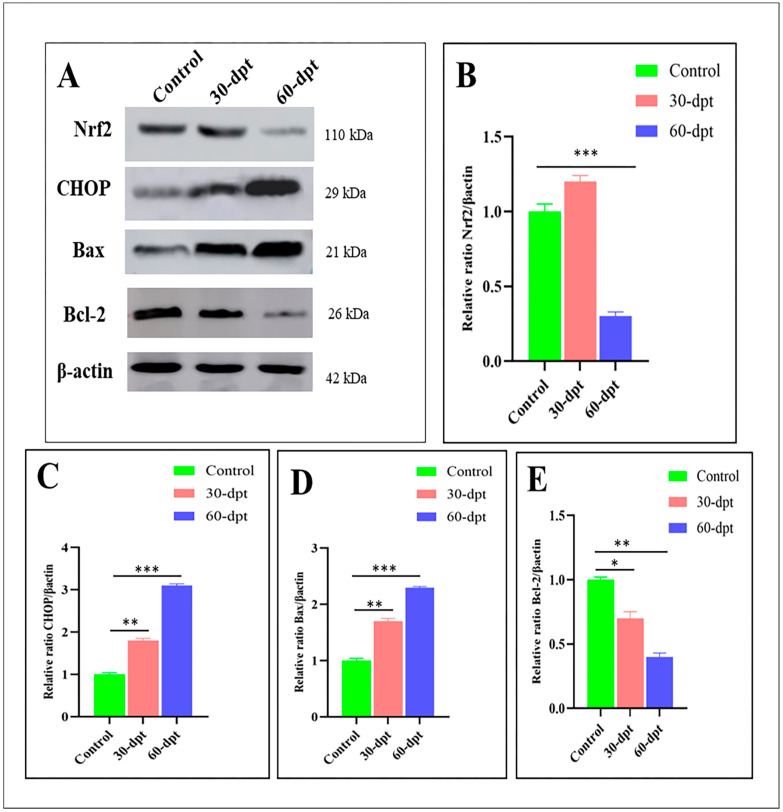
Apoptotic pathway induction by ZnO-NPs via oxidative stress induction: (**A**) immunoblots showing Nrf2, CHOP, Bax, and Bcl-2 proteins of control, 30-dpt, and 60-dpt (β-actin was used for normalization and relative protein levels were quantified using NIH software Image J), (**B**–**E**) protein expression revealing the ratio analysis of Nrf2/βactin, CHOP/βactin, BAX/β actin, Bcl-2/βactin quantified by the Image J 1.51k; Java 1.6.0_24 (64-Bit). The results are the mean ± SEM. * *p* < 0.05, ** *p* < 0.01, *** *p* < 0.001.

Western blot analysis was conducted on the control, (30-dpt), and (60-dpt) groups to reveal the dramatic changes in the protein expression for the previously mentioned pathways. Herein, nuclear factor erythroid 2-related factor 2 (Nrf2), a crucial regulator in the cellular defense against oxidative stress, was slightly up-regulated after 30-dpt, indicating the cytoprotective effect and antioxidative response of Nrf2 against ZnO-NPs toxicity. Nrf2 was suddenly down-regulated after 60-dpt, indicating its suppression due to high ROS accumulation (Figure 7A,B). At the same time, CHOP, which is involved in apoptosis via DNA damage and growth arrest [52], exhibited higher expression at 60-dpt (Figure 7A–C). In addition, Bax, a core regulator of the cellular pathway of apoptosis, was highly up-regulated in the 60-dpt group compared with the control or 30-dpt groups (Figure 7A–D). Contrarily, the anti-apoptotic protein, BecL-2, exhibited a lower expression in the 60-dpt group compared with the control or the 30-dpt groups (Figure 7A–E). Altogether, these results ensure the highly oxidative damage in the olfactory epithelium after 60-dpt of ZnO-NPs, leading to DNA damage and cellular apoptosis.

## 4. Discussion

Unlike most other sensory systems, the olfactory organs are externally open to the surrounding environment, allowing exposure to various toxicants [53]. Although the cytotoxicity of ZnO-NPs has been widely studied, the underlying mechanisms are still unclear [24,54,55,56,57,58,59,60]. Olfaction is a critical sense for detecting and discriminating the surrounding environment of all vertebrates, finding food, and recognizing adverse conditions [61]. Generally, the environmental chemical information is transmitted from the olfactory organ to the brain via the olfactory nerve, causing alarm response, predator avoidance, food search, social communication, reproductive activity, and migration [62,63,64].

Zebrafish, as with other animals [65,66], possess a well-developed sense of smell, responsible for various behaviors involved in reproduction, appetite, and fear. Moreover, the functional properties within its sensory epithelium and the olfactory bulb are comparable to those of mammals [67].

Despite the morphological variations and anatomy of the olfactory system within different vertebrates, the neural basis of odor detection is well maintained since the sense of smell is necessary to perform key functions, mainly securing survival [68,69]. Teleost, such as zebrafish, have a sole olfactory epithelium located on the floor of the nasal cavity and lack the separate vomeronasal organs of higher vertebrates [70]. This olfactory epithelium is organized in lamellae, which radiate from a central ridge or raphe and give rise to an olfactory rosette set on the bottom surface of the nasal cavity on each side of the skull [9,71]. From each olfactory sensory cell, outspread axonal projection forms a fasciculate olfactory nerve [72] and a well-organized neuronal circuitry [73]. So, zebrafish is a key model organism for studying the olfactory system [74].

In all teleosts, there are two main types of OSNs: ciliated and microvillous OSNs. These two morphologically distinct receptor cell types have been distributed in differential patterns within the olfactory epithelium since they are the foremost OSNs in the zebrafish [73]. Nonetheless, teleost, in addition to these two major types of OSN, have two more minor OSN types: crypt OSNs, which express only a single olfactory receptor [75], and the recently identified Kappe neurons [76] that are believed to be absent in tetrapods [77].

All four OSN types are recognized by their morphological features like the cell shape and position within the olfactory epithelium, axons, and dendritic features and preferential response to different stimuli [78]. Ciliated OSNs tend to have a very slender pattern and instead basally situated cell bodies with long apical dendrite-bearing cilia usually radiating from an olfactory knob at its distal ends and extending to the surface of the lamellae, as also shown in channel catfish [10,78] and zebrafish [79,80]. On the other side, the microvillous OSNs are located at the intermediate depths of the epithelium. They have short apical microvilli stemming from a thick dendrite. These microvillous neurons are responded to by amino acids, as mentioned in Salmonids [81], Rainbow trout [82], channel catfish [78], and zebrafish [79,83]. In other words, the ciliated OSNs serve as generalists, whereas microvillous are specialists. Salmonids and cyprinids have shown that both ciliated and microvillous OSNs generally respond to amino acid odorants, but bile acids stimulate ciliated OSNs, and nucleotides activate microvillous OSNs [71]. Earlier, Cancalon (1982) clarified that zinc could induce an extensive degeneration of the olfactory sensory cells and result in severe damage [84].

Exposure to mercury, a toxic and harmful metal for the olfactory organs, reacted differently with the three main types of OSNs. Bazáes and Schmachtenberg (2012) recently showed that microvillous OSNs are more susceptible to mercury exposure than ciliated OSNs, and crypt cell density decreases [85].

Crypt neurons constitute an exciting cell population comprising the third type of olfactory receptor neurons. They appeared early in vertebrate evolution since they are common in almost all marine and freshwater actinopterygian fish, even in cartilaginous fish, but not sarcopterygian lungfishes [77,86]. Such cells are observed by TEM only at the olfactory lamellar surface, which was probably due to their positioning and enwrapping between other closely adjacent receptors and supporting cells [63]. Crypt neurons are located close to the epithelial surface in addition to relatively short microvilli and submerged cilia without olfactory knobs [74,85]. Many attempts have been made to clarify the function of the crypt cell during spawning, indicating its significant relation to the reproductive status and sex pheromone detectors [87].

Conversely, Kappe neurons are a limited cell population exhibiting conspicuous short globular shape somata equipped with a few cilia and microvilli. They are positioned superficially within the olfactory epithelium close to the lumen and far away from the basal lamina [75]. Ahuja et al. (2015) suggested that they are new types of OSNs characterized by their cap apical end [76].

Their absolute existence in the olfactory epithelium shows an astonishing complexity of odor signs already in the periphery of the olfactory system. In addition, the present study described the pear-shaped cell, which is located in the apical part of the epithelium, with numerous mitochondria, ribosomes, and extremely short dendrites, and this is in agreement with other authors, who declared that these cells are present in a very small number in the most superficial layer of the olfactory epithelium and serving for detection of adenosine [73,88].

Cheung et al. (2021) later confirmed the presence of a rare rod cell in the olfactory epithelium of zebrafish, especially in the early stages [89]. They are morphologically distinguishing from the other well-known sensory cells as they possess a conspicuous single compound cilium-like-rod extending from the center of a knob-like apex without a detectable axon. So, they do not belong to the two broad classes of sensory receptors, ciliated and microvillous, but have been identified based on morphology, receptor expression, and projection pattern [90]. Olfactory rod cells have been previously described in many other teleost fish species, including several eel species [91], goldfish [92], rainbow trout [93], common bleak [94], catfish [95], *Mugil cephalus,* and *Malapterurus electricus* [96] cave loach species [97,98]. However, some authors presumed that the existence of receptor cells bearing rod cilium or microvilli represented intermediate stages of the ciliated one [99,100].

Interestingly, rod cells are more distinctive from rodlet cells, described in many different epithelial tissues of all fish, including zebrafish [101,102]. The rodlet cells appeared in the olfactory epithelia of the presently studied zebrafish, often among sensory and non-sensory epithelia. These are ovoid-shaped cells enclosed by a distinctive thick cuticula-like wall and have been reported as being immature stages of goblet cells [103].

Nonetheless, these cells, initially supposed to be protozoan parasites, are prevalent in fish organ tissue and are not parasitic [104,105,106]. These confusing rodlet cells are often associated with fish viscera and epithelium such as skin, intestine, kidney tubules, and biliary duct [105,107,108,109,110]. These rodlet cells represent inflammatory cells closely related to other innate immune cells and potential biomarkers for stressors and chemical agents [111].

Among other nanoparticles, zinc oxide might enhance the odorant responses of OSNs [112]. However, in the present investigation, the cilia, microvilli, and rod of all olfactory ciliated microvilli and rod cells were adversely affected by ZnO-NPs. These dendritic projections were atrophied, minimized in number, and significantly injured. The mitochondria in all these sensory neurons swelled or became filamentous and showed distinctive signs of degeneration compared to the normal, non-treated fish, particularly in the rod cells. Nonetheless, although the crypt cells have both cilia and microvilli not directly immersed in the water flow in the olfactory chamber [113], these were less affected by zinc nanoparticles. Crypt cells’ density dropped and lost their distinctive shape, nuclei shrunk, and their mitochondria were elongated, becoming filamentous, according to other literature reports [114,115]. Alongside, the cilia and micro-ridges of ciliated non-sensory cells were shortened, minimized, or erased.

Furthermore, their surrounding supporting cells were also squeezed and decreased in size [116]. Nonetheless, the goblet mucous cells increased in number and became hyper-activated. Their mucous secretions covered the surface of all the olfactory epithelium with retention of large amounts of ZnO-NPs, as reported by Ghosh et al. (2014), who observed the same effect upon mercury exposure and interpreted it as a type of protection [117]. When the mucous layer becomes excessive, it can prevent pollutants from directly contacting the epithelial surface and decrease toxicity effects [118].

Accordingly, ZnO-NPs in the present study induced mitochondrial damage and impaired the genetic antioxidant enzymes synthesized at the genetic level, releasing ROS that modulate oxidative stress, which agrees with previous studies [27,59,119,120]. The super oxidase dismutase (sod1) and (sod2), catalase (cat), and glutathione peroxidase (gpx) are important markers in defending cells against oxidative stress by catalyzing the conversion of superoxide free radicals (O2^−^) into hydrogen peroxide then reduced to water [121,122,123]. Earlier, it was found that ROS can cause severe damage to DNA molecules [124,125,126,127,128], and this was ensured by the up-regulation of cyclin-dependent kinase inhibitor p21, tumor protein p53, and growth arrest gadd45aa expressions indicating the cell cycle arrest after DNA damage [56,129,130,131,132,133]. As a result, damaged DNA activates apoptosis via the loss of membrane integrity, cytoplasmic content fragmentation, and chromatin condensation, as demonstrated earlier [134,135,136].

Nrf2 is a transcription factor activated in response to stress. It significantly enhances the antioxidant defense of stressed cells and helps detoxify and eliminate exogenous chemicals and their toxic metabolites [137]. Herein, Nrf2 was down-regulated after 60-dpt, which might be a defense mechanism against ROS accumulation. Moreover, in the 60-dpt group, there was an up-regulation of the apoptotic CHOP and Bax expression and a down-regulation of the anti-apoptotic protein BcL-2 expression, confirming the apoptotic effect of ZnO-NPs. Similar to these findings, in zebrafish embryos exposed to ZnO-NPs, the gene expression analysis of several genes of antioxidant proteins (Bcl-2, Nqo1, and Gstp2) revealed an important down-regulation [54].

Furthermore, the production of ROS, which is the basis of oxidative stress, was confirmed by an altered expression of anti-apoptotic genes (Bcl-2, B-cell lymphoma2) and pro-apoptotic (bax, puma, and apaf-1) genes in zebrafish exposed to ZnO-NPs, where the expression of BCL2-associated X apoptosis regulator (Bax) was significantly up-regulated, while Bcl-2 was down-regulated [138]. The overexpression of CHOP and microinjection of CHOP protein have been reported to lead to cell cycle arrest and/or apoptosis [139]. Moreover, the overexpression of CHOP leads to decreased Bcl-2 protein and translocation of Bax protein from the cytosol to the mitochondria [140].

## 5. Conclusions

The present study shows that ZnO-NPs can mediate the cellular oxidative stress and arrest cell growth that induces apoptosis without the ability of cellular regeneration, hence leading to sensory toxicity that directly damages the olfactory epithelium and affects fish smell.

## Figures and Tables

**Table 1:** Primer sequences (forward and reverse) used for real-time qPCR analysis.

Gene	Name	Primers	Accession (Gene ID)
*sod1*	Superoxide dismutase1	F: 5′ CGCACTTCAACCCTCATGAC 3′ R: 5′ TGAATCACCATGGTCCTCCC 3′	NM_131294
*sod2*	Superoxide dismutase2	F:5’ CCTCCAGACAGAAGCA 3’ R:5’ CTGAAATGAGCCAAAGT 3’	NM_199976
*gpx1a*	glutathione peroxidase 1a	F:5’ GCACAACAGTCAGGGAT 3’ R:5’ TCAGGAACGCAAACAG 3’	NM_001007281
*gstp1.2*	glutathione S-transferase pi 1.2	F: 5′ CCAACCACCTCAAATGCT 3′ R: 5′ ACGGGAAAGAGTCCAGACAG 3′	NM_131734
*cat*	Catalase	F: 5’ TGTGGAAGGAGGGTCG 3’ R: 5′ CTTTGGCTTTGGAGTAG 3′	NM_130912
*p21*	cyclin-dependent kinase inhibitor 1A (cdkn1a)	F: 5′ CCTACGTTCACTCGGTAATGGG 3′ R: 5′ CACTAGACGCTTCTTGGCTTGG 3′	NM_001128420
*p53*	Tumor protein p53 (tp53), transcript variant 2	F: 5′ GCAGTCTGGCACAGCAAAATCTGT 3′ R: 5′ TCAGCCACATGCTCGGACTTCTTA 3′	NM_131327
*gadd45aa*	Growth arrest and DNA-damage-inducible, alpha, a	F: 5′ GCTGCGAGAACGACATCAACA 3′ R: 5′ GGGCACCCACTGATCCATACA 3′	NM_200576
*siva1*	Apoptosis-inducing factor	F: 5′ CCGCTACCGACAGGAGATCTACGA 3′ R: 5′ GGTGTGGAGCGCGCTCTGTGCAGT 3′	NM_001327928
*baxa*	BCL2 associated X, apoptosis regulator	F: 5 GACAGGGATGCTGAAGTGA 3′ R: 5′ TGAGTCGGCTGAAGATTAGA 3′	NM_131562
*caspa*	caspase a (caspa)	F: 5′ GACGGTGAGCCTGATGAGCCAA 3′ R: 5′ CCTGAACAGTTCCTCGATGTGA 3′	NM_131505
*Actin b1*	actin, beta 1 (actb1)	F: 5’ ATGGATGAGGAAATCGCTGC 3’ R:5’ CTTTCTGTCCCATGCCAACC 3’	NM_131031

## Data Availability

The data presented in this study are available on request from the corresponding authors.

## References

[1] Keller A.A., McFerran S., Lazareva A., Suh S. (2013). Global life cycle releases of engineered nanomaterials. J. Nanoparticle Res..

[2] Piccinno F., Gottschalk F., Seeger S., Nowack B. (2012). Industrial production quantities and uses of ten engineered nanomaterials in Europe and the world. J. Nanoparticle Res..

[3] Di Cerbo A., Pezzuto F., Scarano A. (2016). Cytotoxic and Bacteriostatic Activity of Nanostructured TiO_2_ Coatings. Pol. J. Microbiol..

[4] Di Cerbo A., Mescola A., Iseppi R., Canton R., Rossi G., Stocchi R., Loschi A.R., Alessandrini A., Rea S., Sabia C. (2020). Antibacterial Effect of Aluminum Surfaces Untreated and Treated with a Special Anodizing Based on Titanium Oxide Approved for Food Contact. Biology.

[5] Di Cerbo A., Rosace G., Rea S., Stocchi R., Morales-Medina J.C., Canton R., Mescola A., Condo C., Loschi A.R., Sabia C. (2021). Time-Course Study of the Antibacterial Activity of an Amorphous SiO(_x_)C(_y_)H(_z_) Coating Certified for Food Contact. Antibiotics.

[6] Di Cerbo A., Mescola A., Rosace G., Stocchi R., Rossi G., Alessandrini A., Preziuso S., Scarano A., Rea S., Loschi A.R. (2021). Antibacterial Effect of Stainless Steel Surfaces Treated with a Nanotechnological Coating Approved for Food Contact. Microorganisms.

[7] Berube D.M. (2008). Rhetorical gamesmanship in the nano debates over sunscreens and nanoparticles. J. Nanoparticle Res..

[8] Jiang J., Pi J., Cai J. (2018). The Advancing of Zinc Oxide Nanoparticles for Biomedical Applications. Bioinorg. Chem. Appl..

[9] Wiesmann N., Mendler S., Buhr C.R., Ritz U., Kammerer P.W., Brieger J. (2021). Zinc Oxide Nanoparticles Exhibit Favorable Properties to Promote Tissue Integration of Biomaterials. Biomedicines.

[10] Zhang Y., Nayak T.R., Hong H., Cai W. (2013). Biomedical applications of zinc oxide nanomaterials. Curr. Mol. Med..

[11] Ahamed M., Khan M.A.M., Akhtar M.J., Alhadlaq H.A., Alshamsan A. (2016). Role of Zn doping in oxidative stress mediated cytotoxicity of TiO_2_ nanoparticles in human breast cancer MCF-7 cells. Sci. Rep..

[12] Franklin N.M., Rogers N.J., Apte S.C., Batley G.E., Gadd G.E., Casey P.S. (2007). Comparative Toxicity of Nanoparticulate ZnO, Bulk ZnO, and ZnCl_2_ to a Freshwater Microalga (*Pseudokirchneriella subcapitata*): The Importance of Particle Solubility. Environ. Sci. Technol..

[13] Kumar N., Chandan N.K., Wakchaure G.C., Singh N.P. (2020). Synergistic effect of zinc nanoparticles and temperature on acute toxicity with response to biochemical markers and histopathological attributes in fish. Comp. Biochem. Physiol. C Toxicol. Pharmacol..

[14] Lin D., Xing B. (2007). Phytotoxicity of nanoparticles: Inhibition of seed germination and root growth. Environ. Pollut..

[15] Park S., Lee Y.K., Jung M., Kim K.H., Chung N., Ahn E.K., Lim Y., Lee K.H. (2007). Cellular toxicity of various inhalable metal nanoparticles on human alveolar epithelial cells. Inhal. Toxicol..

[16] Sardoiwala M.N., Kaundal B., Choudhury S.R. (2018). Toxic impact of nanomaterials on microbes, plants and animals. Environ. Chem. Lett..

[17] Zhang L., Jiang Y., Ding Y., Povey M., York D. (2007). Investigation into the antibacterial behaviour of suspensions of ZnO nanoparticles (ZnO nanofluids). J. Nanoparticle Res..

[18] Khalil S.R., Zheng C., Abou-Zeid S.M., Farag M.R., Elsabbagh H.S., Siddique M.S., Azzam M.M., Di Cerbo A., Elkhadrawey B.A. (2023). Modulatory effect of thymol on the immune response and susceptibility to *Aeromonas hydrophila* infection in Nile tilapia fish exposed to zinc oxide nanoparticles. Aquat. Toxicol..

[19] Mawed S.A., Centoducati G., Farag M.R., Alagawany M., Abou-Zeid S.M., Elhady W.M., El-Saadony M.T., Di Cerbo A., Al-Zahaby S.A. (2022). *Dunaliella salina* Microalga Restores the Metabolic Equilibrium and Ameliorates the Hepatic Inflammatory Response Induced by Zinc Oxide Nanoparticles (ZnO-NPs) in Male Zebrafish. Biology.

[20] Di Cerbo A., Canello S., Guidetti G., Fiore F., Corsi L., Rubattu N., Testa C., Cocco R. (2018). Adverse food reactions in dogs due to antibiotic residues in pet food: A preliminary study. Vet. Ital..

[21] Arunachalam M., Raja M., Vijayakumar C., Malaiammal P., Mayden R.L. (2013). Natural history of zebrafish (*Danio rerio*) in India. Zebrafish.

[22] Schilling T.F., Webb J. (2007). Considering the zebrafish in a comparative context. J. Exp. Zool. B Mol. Dev. Evol..

[23] Becker C.G., Becker T. (2008). Adult zebrafish as a model for successful central nervous system regeneration. Restor. Neurol. Neurosci..

[24] Choi J.S., Kim R.O., Yoon S., Kim W.K. (2016). Developmental Toxicity of Zinc Oxide Nanoparticles to Zebrafish (*Danio rerio*): A Transcriptomic Analysis. PLoS ONE.

[25] Kteeba S.M., El-Ghobashy A.E., El-Adawi H.I., El-Rayis O.A., Sreevidya V.S., Guo L., Svoboda K.R. (2018). Exposure to ZnO nanoparticles alters neuronal and vascular development in zebrafish: Acute and transgenerational effects mitigated with dissolved organic matter. Environ. Pollut..

[26] Brooker R.M., Dixson D.L. (2016). Assessing the Role of Olfactory Cues in the Early Life History of Coral Reef Fish: Current Methods and Future Directions. Chemical Signals in Vertebrates 13.

[27] Kondo K., Kikuta S., Ueha R., Suzukawa K., Yamasoba T. (2020). Age-Related Olfactory Dysfunction: Epidemiology, Pathophysiology, and Clinical Management. Front. Aging Neurosci..

[28] Attems J., Walker L., Jellinger K.A. (2015). Olfaction and Aging: A Mini-Review. Gerontology.

[29] Hummel T., Whitcroft K.L., Andrews P., Altundag A., Cinghi C., Costanzo R.M., Damm M., Frasnelli J., Gudziol H., Gupta N. (2016). Position paper on olfactory dysfunction. Rhinology.

[30] Liu B., Luo Z., Pinto J.M., Shiroma E.J., Tranah G.J., Wirdefeldt K., Fang F., Harris T.B., Chen H. (2019). Relationship Between Poor Olfaction and Mortality among Community-Dwelling Older Adults: A Cohort Study. Ann. Intern. Med..

[31] Schubert C.R., Fischer M.E., Pinto A.A., Klein B.E.K., Klein R., Tweed T.S., Cruickshanks K.J. (2017). Sensory Impairments and Risk of Mortality in Older Adults. J. Gerontol. Ser. A Biomed. Sci. Med. Sci..

[32] Razmara P., Lari E., Mohaddes E., Zhang Y., Goss G.G., Pyle G.G. (2019). The effect of copper nanoparticles on olfaction in rainbow trout (*Oncorhynchus mykiss*). Environ. Sci. Nano.

[33] Bilberg K., Døving K.B., Beedholm K., Baatrup E. (2011). Silver nanoparticles disrupt olfaction in Crucian carp (*Carassius carassius*) and Eurasian perch (*Perca fluviatilis*). Aquat. Toxicol..

[34] Gao L., Yang S.T., Li S., Meng Y., Wang H., Lei H. (2013). Acute toxicity of zinc oxide nanoparticles to the rat olfactory system after intranasal instillation. J. Appl. Toxicol..

[35] Westerfield M. (2000). The Zebrafish Book: A Guide for the Laboratory Use of Zebrafish (Danio rerio).

[36] Mawed S.A., Marini C., Alagawany M., Farag M.R., Reda R.M., El-Saadony M.T., Elhady W.M., Magi G.E., Di Cerbo A., El-Nagar W.G. (2022). Zinc Oxide Nanoparticles (ZnO-NPs) Suppress Fertility by Activating Autophagy, Apoptosis, and Oxidative Stress in the Developing Oocytes of Female Zebrafish. Antioxidants.

[37] Ruzhinskaya N.N., Gdovskii P.A., Devitsina G.V. (2001). Chloride Cells, A Constituent of the Fish Olfactory Epithelium. J. Evol. Biochem. Physiol..

[38] Slaoui M., Fiette L. (2011). Histopathology procedures: From tissue sampling to histopathological evaluation. Drug Saf. Eval. Methods Protoc..

[39] Di Cerbo A., Canello S., Guidetti G., Laurino C., Palmieri B. (2014). Unusual antibiotic presence in gym trained subjects with food intolerance; a case report. Nutr. Hosp..

[40] Di Cerbo A., Palmieri B. (2015). Review: The market of probiotics. Pak. J. Pharm. Sci..

[41] Mazzeranghi F., Zanotti C., Di Cerbo A., Verstegen J.P., Cocco R., Guidetti G., Canello S. (2017). Clinical efficacy of nutraceutical diet for cats with clinical signs of cutaneus adverse food reaction (CAFR). Pol. J. Vet. Sci..

[42] Di Cerbo A., Rubino V., Morelli F., Ruggiero G., Landi R., Guidetti G., Canello S., Terrazzano G., Alessandrini A. (2018). Mechanical phenotyping of K562 cells by the Micropipette Aspiration Technique allows identifying mechanical changes induced by drugs. Sci. Rep..

[43] Di Cerbo A., Palmieri B. (2012). The economic impact of second opinion in pathology. Saudi Med. J..

[44] Iannitti T., Palmieri B., Aspiro A., Di Cerbo A. (2014). A preliminary study of painless and effective transdermal botulinum toxin A delivery by jet nebulization for treatment of primary hyperhidrosis. Drug Des. Dev. Ther..

[45] Destefanis S., Giretto D., Muscolo M.C., Di Cerbo A., Guidetti G., Canello S., Giovazzino A., Centenaro S., Terrazzano G. (2016). Clinical evaluation of a nutraceutical diet as an adjuvant to pharmacological treatment in dogs affected by Keratoconjunctivitis sicca. BMC Vet. Res..

[46] Farag M.R., Zizzadoro C., Alagawany M., Abou-Zeid S.M., Mawed S.A., El Kholy M.S., Di Cerbo A., Azzam M.M., Mahdy E.A.A., Khedr M.H.E. (2023). In ovo protective effects of chicoric and rosmarinic acids against Thiacloprid-induced cytotoxicity, oxidative stress, and growth retardation on newly hatched chicks. Poult. Sci..

[47] Farag M.R., Abo-Al-Ela H.G., Alagawany M., Azzam M.M., El-Saadony M.T., Rea S., Di Cerbo A., Nouh D.S. (2023). Effect of Quercetin Nanoparticles on Hepatic and Intestinal Enzymes and Stress-Related Genes in Nile Tilapia Fish Exposed to Silver Nanoparticles. Biomedicines.

[48] Farag M.R., Moselhy A.A.A., El-Mleeh A., Aljuaydi S.H., Ismail T.A., Di Cerbo A., Crescenzo G., Abou-Zeid S.M. (2021). Quercetin Alleviates the Immunotoxic Impact Mediated by Oxidative Stress and Inflammation Induced by Doxorubicin Exposure in Rats. Antioxidants.

[49] Farag M.R., Khalil S.R., Zaglool A.W., Hendam B.M., Moustafa A.A., Cocco R., Di Cerbo A., Alagawany M. (2021). Thiacloprid Induced Developmental Neurotoxicity via ROS-Oxidative Injury and Inflammation in Chicken Embryo: The Possible Attenuating Role of Chicoric and Rosmarinic Acids. Biology.

[50] Gallo A., Landi R., Rubino V., Di Cerbo A., Giovazzino A., Palatucci A.T., Centenaro S., Guidetti G., Canello S., Cortese L. (2017). Oxytetracycline induces DNA damage and epigenetic changes: A possible risk for human and animal health?. PeerJ.

[51] Guildford A.L., Poletti T., Osbourne L.H., Di Cerbo A., Gatti A.M., Santin M. (2009). Nanoparticles of a different source induce different patterns of activation in key biochemical and cellular components of the host response. J. R. Soc. Interface.

[52] Lebeaupin C., Vallee D., Hazari Y., Hetz C., Chevet E., Bailly-Maitre B. (2018). Endoplasmic reticulum stress signalling and the pathogenesis of non-alcoholic fatty liver disease. J. Hepatol..

[53] Hentig J.T., Byrd-Jacobs C.A. (2016). Exposure to Zinc Sulfate Results in Differential Effects on Olfactory Sensory Neuron Subtypes in Adult Zebrafish. Int. J. Mol. Sci..

[54] Bai W., Zhang Z., Tian W., He X., Ma Y., Zhao Y., Chai Z. (2010). Toxicity of zinc oxide nanoparticles to zebrafish embryo: A physicochemical study of toxicity mechanism. J. Nanoparticle Res..

[55] Du J., Wang S., You H., Jiang R., Zhuang C., Zhang X. (2016). Developmental toxicity and DNA damage to zebrafish induced by perfluorooctane sulfonate in the presence of ZnO nanoparticles. Environ. Toxicol..

[56] Hou J., Liu H., Zhang S., Liu X., Hayat T., Alsaedi A., Wang X. (2019). Mechanism of toxic effects of Nano-ZnO on cell cycle of zebrafish (*Danio rerio*). Chemosphere.

[57] Kteeba S.M., El-Adawi H.I., El-Rayis O.A., El-Ghobashy A.E., Schuld J.L., Svoboda K.R., Guo L. (2017). Zinc oxide nanoparticle toxicity in embryonic zebrafish: Mitigation with different natural organic matter. Environ. Pollut..

[58] Lee G., Lee B., Kim K.-T. (2021). Mechanisms and effects of zinc oxide nanoparticle transformations on toxicity to zebrafish embryos. Environ. Sci. Nano.

[59] Zhao X., Wang S., Wu Y., You H., Lv L. (2013). Acute ZnO nanoparticles exposure induces developmental toxicity, oxidative stress and DNA damage in embryo-larval zebrafish. Aquat. Toxicol..

[60] Zhu X., Wang J., Zhang X., Chang Y., Chen Y. (2009). The impact of ZnO nanoparticle aggregates on the embryonic development of zebrafish (*Danio rerio*). Nanotechnology.

[61] Wyatt T.D. (2010). Pheromones and signature mixtures: Defining species-wide signals and variable cues for identity in both invertebrates and vertebrates. J. Comp. Physiol. A Neuroethol. Sens. Neural. Behav. Physiol..

[62] Eisthen H.L. (1997). Evolution of vertebrate olfactory systems. Brain Behav. Evol..

[63] Hamdani E.H., Doving K.B. (2006). Specific projection of the sensory crypt cells in the olfactory system in crucian carp, *Carassius carassius*. Chem. Senses.

[64] Døving K., Kasumyan A. (2008). Chemoreception. Fish Larval Physiology.

[65] Di Cerbo A., Sechi S., Canello S., Guidetti G., Fiore F., Cocco R. (2017). Behavioral Disturbances: An Innovative Approach to Monitor the Modulatory Effects of a Nutraceutical Diet. J. Vis. Exp..

[66] Sechi S., Di Cerbo A., Canello S., Guidetti G., Chiavolelli F., Fiore F., Cocco R. (2017). Effects in dogs with behavioural disorders of a commercial nutraceutical diet on stress and neuroendocrine parameters. Vet. Rec..

[67] Kermen F., Franco L., Wyatt C., Yaksi E. (2013). Neural circuits mediating olfactory-driven behavior in fish. Front. Neural Circuits.

[68] Biechl D., Tietje K., Gerlach G., Wullimann M.F. (2016). Crypt cells are involved in kin recognition in larval zebrafish. Sci. Rep..

[69] Lazzari M., Bettini S., Milani L., Maurizii M.G., Franceschini V. (2017). Differential response of olfactory sensory neuron populations to copper ion exposure in zebrafish. Aquat. Toxicol..

[70] Hansen A., Zeiske E. (1998). The peripheral olfactory organ of the zebrafish, *Danio rerio*: An ultrastructural study. Chem. Senses.

[71] Hansen A., Zielinski B.S. (2005). Diversity in the olfactory epithelium of bony fishes: Development, lamellar arrangement, sensory neuron cell types and transduction components. J. Neurocytol..

[72] Miyasaka N., Wanner A.A., Li J., Mack-Bucher J., Genoud C., Yoshihara Y., Friedrich R.W. (2013). Functional development of the olfactory system in zebrafish. Mech. Dev..

[73] Olivares J., Schmachtenberg O. (2019). An update on anatomy and function of the teleost olfactory system. PeerJ.

[74] Calvo-Ochoa E., Byrd-Jacobs C.A. (2019). The Olfactory System of Zebrafish as a Model for the Study of Neurotoxicity and Injury: Implications for Neuroplasticity and Disease. Int. J. Mol. Sci..

[75] Gayoso J., Castro A., Anadon R., Manso M.J. (2012). Crypt cells of the zebrafish *Danio rerio* mainly project to the dorsomedial glomerular field of the olfactory bulb. Chem. Senses.

[76] Ahuja G., Nia S.B., Zapilko V., Shiriagin V., Kowatschew D., Oka Y., Korsching S.I. (2014). Kappe neurons, a novel population of olfactory sensory neurons. Sci. Rep..

[77] Hansen A., Finger T.E. (2000). Phyletic distribution of crypt-type olfactory receptor neurons in fishes. Brain Behav Evol.

[78] Hansen A., Rolen S.H., Anderson K., Morita Y., Caprio J., Finger T.E. (2003). Correlation between olfactory receptor cell type and function in the channel catfish. J. Neurosci..

[79] Lipschitz D.L., Michel W.C. (2002). Amino acid odorants stimulate microvillar sensory neurons. Chem. Senses.

[80] Sato Y., Miyasaka N., Yoshihara Y. (2005). Mutually exclusive glomerular innervation by two distinct types of olfactory sensory neurons revealed in transgenic zebrafish. J. Neurosci..

[81] Hara T.J., Zhang C. (1998). Topographic bulbar projections and dual neural pathways of the primary olfactory neurons in salmonid fishes. Neuroscience.

[82] Sato K., Suzuki N. (2001). Whole-cell response characteristics of ciliated and microvillous olfactory receptor neurons to amino acids, pheromone candidates and urine in rainbow trout. Chem. Senses.

[83] Oka Y., Korsching S.I. (2011). Shared and unique G alpha proteins in the zebrafish versus mammalian senses of taste and smell. Chem. Senses.

[84] Cancalon P. (1982). Degeneration and regeneration of olfactory cells induced by ZnSO_4_ and other chemicals. Tissue Cell.

[85] Bazaes A., Schmachtenberg O. (2012). Odorant tuning of olfactory crypt cells from juvenile and adult rainbow trout. J. Exp. Biol..

[86] Ferrando S., Bottaro M., Gallus L., Girosi L., Vacchi M., Tagliafierro G. (2006). Observations of crypt neuron-like cells in the olfactory epithelium of a cartilaginous fish. Neurosci. Lett..

[87] Hamdani E.H., Lastein S., Gregersen F., Doving K.B. (2008). Seasonal variations in olfactory sensory neurons—Fish sensitivity to sex pheromones explained?. Chem. Senses.

[88] Wakisaka N., Miyasaka N., Koide T., Masuda M., Hiraki-Kajiyama T., Yoshihara Y. (2017). An Adenosine Receptor for Olfaction in Fish. Curr. Biol..

[89] Cheung K.Y., Jesuthasan S.J., Baxendale S., van Hateren N.J., Marzo M., Hill C.J., Whitfield T.T. (2021). Olfactory Rod Cells: A Rare Cell Type in the Larval Zebrafish Olfactory Epithelium with a Large Actin-Rich Apical Projection. Front. Physiol..

[90] Elsaesser R., Paysan J. (2007). The sense of smell, its signalling pathways, and the dichotomy of cilia and microvilli in olfactory sensory cells. BMC Neurosci..

[91] Yamamoto M., Hara T.J. (1982). Comparative Morphology of the Peripheral Olfactory Organ in Teleosts. Chemoreception in Fishes.

[92] Ichikawa M., Ueda K. (1977). Fine structure of the olfactory epithelium in the goldfish, Carassius auratus. Cell Tissue Res..

[93] Rhein L.D., Cagan R.H., Orkand P.M., Dolack M.K. (1981). Surface specializations of the olfactory epithelium of rainbow trout, Salmo gairdneri. Tissue Cell.

[94] Hernadi L. (1993). Fine structural characterization of the olfactory epithelium and its response to divalent cations Cd^2+^ in the fish Alburnus alburnus (Teleostei, Cyprinidae): A scanning and transmission electron microscopic study. Neurobiology.

[95] Datta N.C., Bandopadhyay S. (1997). Ultrastructure of cell types of the olfactory epithelium in a catfish, *Heteropneustesfossilis* (Bloch). J. Biosci..

[96] Ismail M.H., Salem M.A., Nassar S.A., Mawed S.A. (2013). Comparative morphological and histological studies on the olfactory organ in two models of bony fishes (*Mugil cephalus* and *Malapterurus electricus*): Light and electron microscopic studies. Egypt. J. Zool..

[97] Waryani B., Zhao Y., Zhang C., Abbasi A.R., Ferrando S., Dai R., Soomro A.N., Baloch W.A., Abbas G. (2015). Surface architecture of the olfactory epithelium of two Chinese cave loaches (Cypriniformes: Nemacheilidae: *Oreonectes*). Ital. J. Zool..

[98] Zhang X.Y., Huang Z.Q., Ning T., Xiang X.H., Li C.Q., Chen S.Y., Xiao H. (2018). Microscopic and Submicroscopic Gradient Variation of Olfactory Systems among Six *Sinocyclocheilus* Species Living in Different Environments. Zool. Sci..

[99] Zielinski B.S., Hara T.J. (1992). Ciliated and microvillar receptor cells degenerate and then differentiate in the olfactory epithelium of rainbow trout following olfactory nerve section. Microsc. Res. Tech..

[100] Pashchenko N.I., Kasumyan A.O. (2015). Scanning electron microscopy of development of the olfactory organ in ontogeny of grass carp *Ctenopharyngodon idella*. J. Ichthyol..

[101] DePasquale J.A. (2021). Tropomyosin and alpha-actinin in teleost rodlet cells. Acta Zool..

[102] Dezfuli B.S., Capuano S., Simoni E., Previati M., Giari L. (2007). Rodlet cells and the sensory systems in zebrafish (*Danio rerio*). Anat. Rec..

[103] Al-Hussaini A.H. (1964). On the Nature of Certain Pear-Shaped Cells in the Intestinal Epithelium of Fish. Bull. Inst. Egypte.

[104] Grunberg W., Hager G. (1978). Ultrastructural and histochemical aspects of the rodlet cells from the bulbus arteriosus of *Cyprinus carpio* L. (Pisces: Cyprinidae) (author’s transl). Anat. Anzeiger..

[105] Dezfuli B.S., Simoni E., Rossi R., Manera M. (2000). Rodlet cells and other inflammatory cells of *Phoxinus phoxinus* infected with *Raphidascaris acus* (Nematoda). Dis. Aquat. Org..

[106] Blaiotta G., Murru N., Di Cerbo A., Succi M., Coppola R., Aponte M. (2017). Commercially standardized process for probiotic “Italico” cheese production. LWT-Food Sci. Technol..

[107] Dezfuli B.S., Capuano S., Manera M. (1998). A description of rodlet cells from the alimentary canal of *Anguilla anguilla* and their relationship with parasitic helminths. J. Fish Biol..

[108] Fishelson L., Becker K. (1999). Rodlet cells in the head and trunk kidney of the domestic carp (*Cyprinus carpio*): Enigmatic gland cells or coccidian parasites?. Naturwissenschaften.

[109] Iger Y., Abraham M. (1997). Rodlet cells in the epidermis of fish exposed to stressors. Tissue Cell.

[110] Scarano A., Murmura G., Vantaggiato G., Lauritano D., Silvestre-Rangil J., Di Cerbo A., Lorusso F. (2017). Delayed expansion of atrophic mandible (deam): A case report. Oral Implant..

[111] Dezfuli B.S., Pironi F., Maynard B., Simoni E., Bosi G. (2022). Rodlet cells, fish immune cells and a sentinel of parasitic harm in teleost organs. Fish Shellfish Immunol..

[112] Viswaprakash N., Dennis J.C., Globa L., Pustovyy O., Josephson E.M., Kanju P., Morrison E.E., Vodyanoy V.J. (2009). Enhancement of odorant-induced responses in olfactory receptor neurons by zinc nanoparticles. Chem. Senses.

[113] Lazzari M., Bettini S., Ciani F., Franceschini V. (2007). Light and transmission electron microscopy study of the peripheral olfactory organ of the guppy, *Poecilia reticulata* (Teleostei, Poecilidae). Microsc. Res. Tech..

[114] Lazzari M., Bettini S., Milani L., Maurizii M.G., Franceschini V. (2019). Differential nickel-induced responses of olfactory sensory neuron populations in zebrafish. Aquat. Toxicol..

[115] Lazzari M., Bettini S., Milani L., Maurizii M.G., Franceschini V. (2022). Response of Olfactory Sensory Neurons to Mercury Ions in Zebrafish: An Immunohistochemical Study. Microsc. Microanal..

[116] Razmara P., Pyle G.G. (2022). Recovery of rainbow trout olfactory function following exposure to copper nanoparticles and copper ions. Aquat. Toxicol..

[117] Ghosh D., Mandal D.K. (2014). Mercuric chloride induced toxicity responses in the olfactory epithelium of *Labeo rohita* (Hamilton): A light and electron microscopy study. Fish Physiol. Biochem..

[118] Roy D., Ghosh D., Mandal D.K. (2012). Induction of Metallothionein in the Olfactory Epithelium of *Channa punctatus* (Bloch) in Response to Cadmium Exposure: An Immunohistochemical Study. Proc. Zool. Soc..

[119] Saliani M., Jalal R., Goharshadi E. (2016). Mechanism of oxidative stress involved in the toxicity of ZnO nanoparticles against eukaryotic cells. Nanomed. J..

[120] Verma S.K., Panda P.K., Jha E., Suar M., Parashar S.K.S. (2017). Altered physiochemical properties in industrially synthesized ZnO nanoparticles regulate oxidative stress; induce in vivo cytotoxicity in embryonic zebrafish by apoptosis. Sci. Rep..

[121] Wang Y., Branicky R., Noe A., Hekimi S. (2018). Superoxide dismutases: Dual roles in controlling ROS damage and regulating ROS signaling. J. Cell Biol..

[122] Hwang J., Jin J., Jeon S., Moon S.H., Park M.Y., Yum D.Y., Kim J.H., Kang J.E., Park M.H., Kim E.J. (2020). SOD1 suppresses pro-inflammatory immune responses by protecting against oxidative stress in colitis. Redox Biol..

[123] Ighodaro O.M., Akinloye O.A. (2018). First line defence antioxidants-superoxide dismutase (SOD), catalase (CAT) and glutathione peroxidase (GPX): Their fundamental role in the entire antioxidant defence grid. Alex. J. Med..

[124] Preedia Babu E., Subastri A., Suyavaran A., Lokeshwara Rao P., Suresh Kumar M., Jeevaratnam K., Thirunavukkarasu C. (2015). Extracellularly synthesized ZnO nanoparticles interact with DNA and augment gamma radiation induced DNA damage through reactive oxygen species. RSC Adv..

[125] Barzilai A., Yamamoto K. (2004). DNA damage responses to oxidative stress. DNA Repair.

[126] Panda K.K., Golari D., Venugopal A., Achary V.M.M., Phaomei G., Parinandi N.L., Sahu H.K., Panda B.B. (2017). Green Synthesized Zinc Oxide (ZnO) Nanoparticles Induce Oxidative Stress and DNA Damage in *Lathyrus sativus* L. Root Bioassay System. Antioxidants.

[127] Payne C.M., Bernstein C., Bernstein H. (1995). Apoptosis overview emphasizing the role of oxidative stress, DNA damage and signal-transduction pathways. Leuk. Lymphoma.

[128] Di Cerbo A., Pezzuto F., Guidetti G., Canello S., Corsi L. (2019). Tetracyclines: Insights and updates of their use in human and animal pathology and their potential toxicity. Open Biochem. J..

[129] Chen Z., Wan X., Hou Q., Shi S., Wang L., Chen P., Zhu X., Zeng C., Qin W., Zhou W. (2016). GADD45B mediates podocyte injury in zebrafish by activating the ROS-GADD45B-p38 pathway. Cell Death Dis..

[130] Kumar A., Najafzadeh M., Jacob B.K., Dhawan A., Anderson D. (2015). Zinc oxide nanoparticles affect the expression of p53, Ras p21 and JNKs: An ex vivo/in vitro exposure study in respiratory disease patients. Mutagenesis.

[131] Macleod K.F., Sherry N., Hannon G., Beach D., Tokino T., Kinzler K., Vogelstein B., Jacks T. (1995). p53-dependent and independent expression of p21 during cell growth, differentiation, and DNA damage. Genes Dev..

[132] Setyawati M.I., Tay C.Y., Leong D.T. (2013). Effect of zinc oxide nanomaterials-induced oxidative stress on the p53 pathway. Biomaterials.

[133] Ng K.W., Khoo S.P., Heng B.C., Setyawati M.I., Tan E.C., Zhao X., Xiong S., Fang W., Leong D.T., Loo J.S. (2011). The role of the tumor suppressor p53 pathway in the cellular DNA damage response to zinc oxide nanoparticles. Biomaterials.

[134] Norbury C.J., Zhivotovsky B. (2004). DNA damage-induced apoptosis. Oncogene.

[135] Roos W.P., Kaina B. (2006). DNA damage-induced cell death by apoptosis. Trends Mol. Med..

[136] Wang C., Hu X., Gao Y., Ji Y. (2015). ZnO Nanoparticles Treatment Induces Apoptosis by Increasing Intracellular ROS Levels in LTEP-a-2 Cells. BioMed Res. Int..

[137] Lu H., Lei X., Zhang Q. (2015). Moderate activation of IKK2-NF-kB in unstressed adult mouse liver induces cytoprotective genes and lipogenesis without apparent signs of inflammation or fibrosis. BMC Gastroenterol..

[138] Du J., Cai J., Wang S., You H. (2017). Oxidative stress and apotosis to zebrafish (*Danio rerio*) embryos exposed to perfluorooctane sulfonate (PFOS) and ZnO nanoparticles. Int. J. Occup. Med. Environ. Health.

[139] Gotoh T., Oyadomari S., Mori K., Mori M. (2002). Nitric oxide-induced apoptosis in RAW 264.7 macrophages is mediated by endoplasmic reticulum stress pathway involving ATF6 and CHOP. J. Biol. Chem..

[140] McCullough K.D., Martindale J.L., Klotz L.O., Aw T.Y., Holbrook N.J. (2001). Gadd153 sensitizes cells to endoplasmic reticulum stress by down-regulating Bcl2 and perturbing the cellular redox state. Mol. Cell. Biol..

